# Redefining the ‘Facial Danger Zone’: A Meta-Analytic Anatomical Mapping of Safer Injection Corridors in the Perinasal Region

**DOI:** 10.1093/asjof/ojag138.012

**Published:** 2026-07-24

**Authors:** Khalifa Al-Ghanim, Kathy Zhang, Kyle Thompson, Sarah Appleton, John Stein

**Affiliations:** Division of Plastic and Reconstructive Surgery, Western University, Canada; Division of Plastic and Reconstructive Surgery, Western University, Canada; Division of Plastic and Reconstructive Surgery, Western University, Canada; Division of Plastic and Reconstructive Surgery, Western University, Canada; Division of Plastic and Reconstructive Surgery, Western University, Canada

## Abstract

**Background:**

The traditional concept of the “facial danger triangle” has guided conservative filler injection practices due to concerns about retrograde embolization through branches of the ophthalmic artery. However, this model lacks anatomical precision. Advances in cadaveric dissection and imaging now allow for detailed mapping of facial vasculature, enabling a more refined, risk-stratified approach to injection safety. The purpose of this study was to:

1. Systematically synthesize anatomical data from cadaveric and imaging-based studies involving the dorsal nasal artery (DNA), lateral nasal artery (LNA), and superior labial artery (SLA).
2. Generate an evidence-based vascular risk map of the perinasal region using pooled vessel diameter, depth, course, and landmark relationships.
3. Convert these findings into anatomically justified recommendations for safer filler injection corridors.
4. Establish whether a meta-analytic approach can meaningfully inform future medical illustration standards and safe clinical practice.

**Methods:**

A systematic search and meta-analysis were performed incorporating 28 anatomical studies (21 cadaveric, 7 imaging-based) producing 409 extractable vascular data points. Included studies reported vessel diameter, course, depth, length, and proximity to fixed cephalometric or anthropometric landmarks. Data from three vessels of greatest clinical relevance—the DNA, LNA, and SLA—were catalogued.

When possible, pooled estimates were calculated using random-effects models, with heterogeneity assessed using τ² and I². For variables with insufficient data, structured descriptive synthesis (mean, SD, and range) was performed. Additional analyses included bilateral comparison where applicable. Tables and position-based mapping were used to integrate findings into visually interpretable risk-stratified zones. Outcome measures:

- Vessel diameter (mm)
- Vessel depth (mm)
- Course and trajectory relative to landmarks
- Symmetry (side-to-side differences)
- High-risk vs. low-risk injection planes and corridors

This data was used to generate an evidence-derived update to the classical danger triangle and propose anatomically grounded injection recommendations.

**Results:**

**Dorsal Nasal Artery (DNA)**:

**Diameter:** ∼0.68 mm (95% CI 0.62–0.73; I² = 0%).

**Depth:** 1.9–2.1 mm in available imaging studies.

**Position:** ∼7 mm from the medial canthus; superficial near the radix.

**Notable branch:** A bilateral communicating vessel ∼8.5 ± 3.5 mm below intercanthal line.

**Risk zones:** Radix and glabella—superficial, consistent, and highly dangerous.

**Lateral Nasal Artery (LNA)**:

**Diameter:** ∼0.97 mm (95% CI 0.74–1.20; I² = 95.8%).

**Course:** ∼1.15 ± 1.25 mm from alar base.

**Length:** ∼10.6 ± 4.5 mm.

**Origin:** ∼10 ± 6.8 mm from oral commissure.

**Symmetry:** No significant left-right differences (p > 0.25).

**Risk zones:** Lateral upper nose is variable but generally safer than midline; still unpredictable due to high heterogeneity.

**Superior Labial Artery (SLA)**:

**Diameter:** ∼1.38 mm (95% CI 1.16–1.59; I² = 90%).

**Depth:** ∼4.8 mm (95% CI 3.52–6.08; I² = 98.6%).

**Length:** ∼21.3 mm (wide range).

**Course:** Predominantly **intramuscular** at commissure; becomes submucosal centrally.

**Risk zones:** Central upper lip remains high risk; lateral lip and alar base areas are more forgiving.

**Conclusion:**

This comprehensive anatomical meta-analysis demonstrates that the traditional “danger triangle” oversimplifies the vascular reality of the perinasal region. By integrating highquality cadaveric and imaging-based data, we establish a more nuanced, evidence-derived vascular map that identifies consistent high-risk midline structures and reproducible lower-risk lateral corridors. These findings challenge outdated injection guidelines and support a shift toward:

1. **Risk-stratified, anatomy-first injection planning** rather than reliance on broad geometric zones.
2. **Improved anatomical reporting standards**, emphasizing fixed anatomic landmarks.
3. **Better medical illustration tools** grounded in pooled, reproducible data.
4. **Continued but realistic use of ultrasound**, recognizing its limitations in the central face.

Ultimately, safe filler injection requires precise anatomical knowledge, thoughtful technique, and an understanding of variability—not blind trust in any device or outdated diagram. This meta-analysis provides the most comprehensive evidence to date for redefining the facial danger zone and advancing safer perinasal injection practice.

